# Predictors of Early Versus Late Recurrence in Invasive Lobular Carcinoma of the Breast: Impact of Local and Systemic Therapy

**DOI:** 10.1245/s10434-023-13881-x

**Published:** 2023-07-18

**Authors:** Harriet T. Rothschild, Elle N. Clelland, Firdows Mujir, Helena Record, Jasmine Wong, Laura J. Esserman, Michael Alvarado, Cheryl Ewing, Rita A. Mukhtar

**Affiliations:** 1 School of Medicine, University of California San Francisco, San Francisco, USA; 2 Department of Surgery, Division of Surgical Oncology, University of California San Francisco, San Francisco, CA USA

## Abstract

**Background:**

Invasive lobular carcinoma (ILC) of the breast is known for high risk of late recurrence, yet some patients still recur within 5 years of diagnosis. Determining factors associated with early/late recurrence could help tailor treatment and surveillance strategies.

**Methods:**

Using an institutional database, we evaluated patients with ILC and ≥ 5 years of follow-up or recurrence within 5 years. We used multivariate logistic regression and the Kaplan-Meier method to evaluate which clinicopathologic features and treatment strategies were associated with recurrence < 5 years since diagnosis versus recurrence ≥ 5 years since diagnosis. Additionally, we explored the association between Clinical Treatment Score 5 (CTS5) with early versus late recurrence.

**Results:**

Among 513 cases of stage I–III ILC, there were 75 early and 54 late recurrences during a median follow-up period of 9.4 years. Early recurrence was associated with larger tumors (mean 4.2 cm vs. 2.9 cm, *p *< 0.0001), higher incidence of > 3 positive nodes (32.4% vs. 9.11%, *p* > 0.0001), and more aggressive tumor biology (low/negative progesterone receptor expression, higher grade, and higher Ki67). Late recurrence was associated with younger age (mean 55.6 vs. 59.2 years, *p *= 0.037) and elevated body mass index (BMI > 25 kg/m^2^ in 60.1.0% vs. 45.4%, *p *= 0.021). Omission of adjuvant endocrine therapy or radiotherapy after lumpectomy conferred increased risk of early rather than late recurrence.

**Conclusion:**

Factors related to tumor aggressiveness and treatment were associated with early recurrence, whereas patient related factors were related to late recurrence. These data may help guide treatment strategies and surveillance approaches for patients with ILC.

## Background

Invasive lobular carcinoma (ILC) is the second most common subtype of breast cancer, making up 10–15% of all cases, and differs from invasive ductal carcinoma (IDC) in several ways.^1^ Data show that patients with ILC are diagnosed at older ages and at more advanced clinical stage, yet have tumors that tend to be better differentiated and estrogen receptor (ER) positive.^2–4^ Many investigators have noted the propensity for ILC to have late recurrences, with studies showing higher cumulative recurrence risk in patients with ILC compared with those with IDC when longer follow-up times are reported.^1,5,6^ While hormone receptor positivity has been reported to be associated with late recurrence risk, a large prospective cohort study found that patients with ILC had significantly worse late prognosis than IDC, independent of ER status.^7,8^ However, despite this apparent proclivity towards late recurrence, there remain some patients with ILC who experience recurrence within the first 5 years after diagnosis.

Understanding what factors are associated with early versus late recurrence could be beneficial for prognostication, treatment selection, and developing an optimal surveillance strategy. Additionally, for older women with ER positive breast cancer, there is considerable interest in omission of adjuvant endocrine therapy or radiation after breast conserving surgery.^9–11^ How these treatment decisions impact risk and timing of recurrence in patients with ILC specifically is not well understood.

Prior investigators have reported on factors associated with early vs. late recurrence for breast cancer in general, and have described a predictive model of recurrence after 5 years for post-menopausal patients with hormone receptor positive breast cancer, the Clinical Treatment Score 5 (CTS5).^12,13^ Of note, few of the reported studies focus on ILC, and only one other published study evaluated CTS5 in ILC specifically. Conforti et al. found that factors associated with late recurrence in ILC included larger tumor size, positive lymph nodes, and a Ki67 of 20% or higher.^14^ CTS5 score was predictive of late recurrence when combined with Ki67.^14^ Treatment factors such as the impact of adjuvant therapy were not reported.

Given the paucity of data on predicting timing of recurrence in ILC, we sought to identify patient and tumor factors associated with timing of recurrence and, importantly, to evaluate whether omission of adjuvant therapy impacts early vs. late recurrence. Additionally, we evaluated the relationship between CTS5 score and recurrence timing overall, and stratified by menopausal status.

## Methods

### Patient Cohort, Study Design, and Definitions

**Fig. 1:**
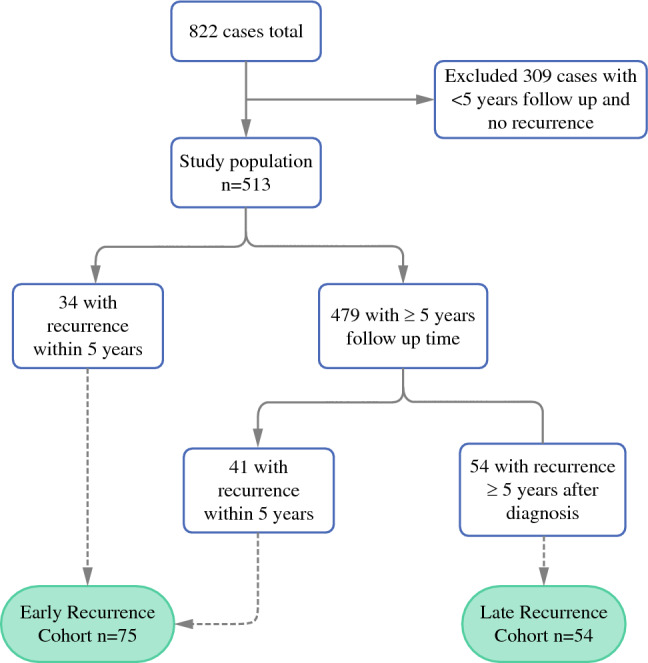
Flow chart of inclusion and exclusion of cases from a prospectively maintained single institution cohort resulting in 75 cases of early recurrence (within 5 years of diagnosis) and 54 cases of late recurrence (after 5 years since diagnosis)

With institutional review board approval, we retrieved clinicopathologic data from a prospectively maintained institutional database containing treatment and outcomes data for ILC patients undergoing surgery at the University of California, San Francisco between January 1996 and September 2019. We included patients with tumors that had lobular or mixed lobular/ductal histology, and were stage I–III at the time of diagnosis (Fig. 1). We excluded patients with less than 5 years of follow-up since the date of diagnosis unless they had a recurrence within the first 5 years. Histologic type, tumor grade, estrogen receptor (ER), progesterone receptor (PR), and Human Epidermal Growth Factor Receptor 2 (HER2) expression were determined from pathology reports. Stage was based on the 7th edition of the American Joint Committee on Cancer Manual.^15^ Tumors with ER staining ≥ 1% on immunohistochemistry (IHC) were considered ER positive, and those with PR staining ≥ 1% on IHC were considered PR positive. We classified “PR low” tumors as those with PR expression of 0–20% on IHC. When available, Ki67 was recorded on a continuous scale, and also dichotomized into < 20% and ≥ 20% positive. Menopausal status at time of diagnosis was obtained from medical oncology notes. Body mass index (BMI) at time of diagnosis was calculated as (weight kg)/(height m^2^), and categorized according to World Health Organization criteria (normal: < 25 kg/m^2^; overweight: 25–30 kg/m^2^; obese: ≥ 30 kg/m^2^).^16^

Recurrence was defined as any invasive recurrence event, including local or distant. We categorized patients into 3 groups defined as follows: those in the “early recurrence” cohort had local or distant recurrence within 5 years of ILC diagnosis; those in the “late recurrence” cohort had their first local or distant recurrence occurring 5 or more years after ILC diagnosis; finally, those in the “no recurrence” group had 5 or more years of follow-up with no recurrence. The presence of recurrence was determined by review of electronic medical records.

We calculated the Clinical Treatment Score at 5 years (CTS5) for all cases with available data. The CTS5 is a validated prognostic tool to estimate distant recurrence risk after 5 years of endocrine therapy for postmenopausal women with ER-positive breast cancer.^13,17,18^ It incorporates information on age (continuous, in years), tumor size (continuous, in cm), quadratic tumor size, nodal status (five groups: 0, negative nodes; 1, one positive node; 2, two to three positive nodes; 3, four to nine positive nodes; and 4, at least nine positive nodes), and grade (three groups: 1, low; 2, intermediate; and 3, high).^14,19^ The formula is CTS5 = 0.438 × nodes + 0.988 × (0.093 × tumor size − 0.001 × (tumor size)^2^ + 0.375 × grade + 0.017 × age).^13,17^ We classified CTS5 score as low, medium, and high, as previously described (low, CTS5 score < 3.13; intermediate, CTS5 score 3.13 to 3.86; and high, CTS5 score > 3.86).^12^

### Statistical Analysis

The primary objective of the study was to identify the clinicopathologic and treatment characteristics associated with early recurrence and late recurrence, respectively, in patients with ILC. Additionally, within the “early recurrence” group, we investigated factors associated with shorter time to early recurrence and used the Kaplan-Meier method to estimate cumulative incidence of recurrence within the first 5 years. Finally, we evaluated whether CTS5 score was associated with early or late recurrence (local or distant) overall and stratified by menopausal status. Data were analyzed in Stata 17 using chi-squared tests, *t*-tests, and multivariate logistic regression models. For time to event analyses, we utilized the log rank test, Kaplan-Meier curves, and multivariate Cox proportional hazard models. Patients without recurrence were censored at the date of last follow-up.

## Results

### Overall Clinicopathologic Characteristics

From 822 consecutive patients with ILC, we identified 513 cases with either recurrence within 5 years, or a minimum of 5 years of follow-up time for analysis (Table 1). Median follow-up time in the overall cohort was 9.4 years. Overall, the mean age at diagnosis was 58.8 years with a standard deviation (SD) of 11.8 years. There were more patients with stage I disease (*n* = 213, 41.7%) compared with stage II (n = 204, 39.8%) or stage III (n = 95, 18.5%). Most tumors were grade 2 (n = 306, 63.0%), and the most common receptor subtype of ILC in this cohort was ER positive, PR positive, HER2 negative (n = 365, 79.4%) (Table 1).

**Table 1:** Patient characteristics of the overall study cohort of patients with invasive lobular carcinoma (ILC), and in those with early or late ILC recurrence

Characteristic	Overall (n = 513)	Early recurrence (n = 75)	Late recurrence (n = 54)
Patient characteristics			
Age at diagnosis [mean years (SD)]	58.8 (11.8)	59.6 (15.8)	55.7 (12.5)
BMI Category, [*n* (%)]			
18.5–25 kg/m^2^	237 (53.1%)	25 (42.4%)	16 (39.0%)
25-30 kg/m^2^	133 (29.8%)	21 (35.6%)	20 (48.8%)
≥ 30 kg/m^2^	76 (17.0%)	13 (22.0%)	5 (12.2%)
Systemic therapy [*n* (%)]			
Any chemotherapy	198 (39.1%)	39 (52.7%)	27 (50.9%)
Neoadjuvant chemotherapy	70 (13.8%)	17 (23.0%)	10 (18.9%)
Adjuvant endocrine therapy	395 (78.1%)	42 (60.0%)	43 (79.6%)
Local therapy [*n* (%)]			
Lumpectomy	81 (15.8%)	19 (25.3%)	4 (7.41%)
Lumpectomy and radiation	149 (29.1%)	15 (20.0%)	18 (33.3%)
Mastectomy	199 (38.9%)	21 (28.0%)	20 (37.0%)
Mastectomy and radiation	83 (16.2%)	20 (26.7%)	12 (22.2%)
Tumor characteristics			
Tumor size [mean cm (SD)]	3.1 (2.9)	4.2 (3.9)	3.3 (2.9)
Positive lymph nodes [*n* (%)]			
0 nodes	341 (67.9%)	33 (44.6%)	29 (55.8%)
1–3 nodes	98 (19.5%)	17 (23.0%)	13 (25.0%)
≥ 3 nodes	63 (12.6%)	24 (32.4%)	10 (19.2%)
Stage [*n* (%)]			
1	214 (41.7%)	18 (24.0%)	20 (37.0%)
2	204 (39.8%)	25 (33.3%)	21 (38.9%)
3	95 (18.5%)	32 (42.7%)	13 (24.1%)
Grade [*n* (%)]^a^			
1	157 (32.3%)	19 (27.9%)	13 (29.6%)
2	306 (63.0%)	36 (52.9%)	29 (65.9%)
3	23 (4.7%)	13 (19.1%)	2 (4.6%)
Ki-67 [mean (SD)]^b^	14.2 (14.3)	21.1 (26.5)	19.8 (16.7)
Lymphovascular invasion [*n* (%)]^c^	41 (8.6%)	14 (21.2%)	4 (9.1%)
Subtype [*n* (%)]^d^			
ER+PR+HER2-	365 (79.4%)	40 (61.5%)	32 (71.1%)
ER+PR-HER2-	56 (12.2%)	14 (21.5%)	6 (13.3%)
ER-PR-HER2-	16 (3.48%)	5 (7.69%)	3 (6.67%)
HER2+	23 (5.00%)	6 (9.23%)	4 (8.89%)
Progesterone receptor [*n* (%)]^e^			
≤ 20% positivity	151 (34.5%)	28 (53.9%)	20 (55.6%)
> 20% positivity	287 (65.5%)	24 (46.2%)	16 (44.5%)
CTS5 scores [*n* (%)]^f^			
Low risk (< 3.13)	162 (34.4%)	15 (23.8%)	10 (23.8%)
Intermediate risk (3.13–3.86)	73 (15.5%)	6 (9.52%)	6 (14.3%)
High risk (> 3.86)	236 (50.1%)	42 (66.7%)	26 (61.9%)

^a^Data available for 486 cases

^b^Data Available for 134 cases

^c^Data available for 479 cases

^d^Data available for 460

^e^Data available for 438 cases

^f^Data available for 722 cases

Total overall n = 513, unless otherwise specified. *BMI* body mass index, *LVI* lymphovascular invasion, *ER* estrogen receptor, *PR* progesterone receptor, *HER2*, Human Epithelial Growth Factor Receptor-2, *CTS5* Clinical Treatment Score 5*.*

Overall, 198 (39.1%) patients received chemotherapy, with 13.8% receiving it neoadjuvantly, and the remaining in the adjuvant setting. Adjuvant endocrine therapy and recurrence data were available for 506 patients; of these, 78.1% received adjuvant endocrine therapy (Table 1). Among the 474 hormone receptor positive cases, adjuvant endocrine therapy was utilized in 382 (80.6%), with 92 cases declining recommended adjuvant endocrine therapy. Surgical treatment was available for 512 subjects, with the most common operation being mastectomy (n = 199, 38.9%). While 149 patients (29.1%) underwent lumpectomy with radiation, 81 patients (15.8%) had omission of adjuvant radiation following lumpectomy.

Of the 513 patients included, there were 75 cases of patients with early recurrence (mean time to recurrence 2.6 years, standard deviation [SD] 1.4), 54 cases of patients with late recurrence (mean time to recurrence 10.1 years, SD 5.8), and 384 cases with no recurrence (mean follow-up time 11.5 years, SD 5.1).

### Clinicopathologic Features Associated with Early Recurrence

Of the 75 patients with early recurrence, 27 (36.0%) had local recurrence, 44 (58.7%) had distant recurrence, and 4 (5.3%) had both local and distant recurrence. Patients with early recurrence had significantly larger tumors compared with the late recurrence and non-recurrence cases (mean ILC size 4.2 cm vs. 2.9 cm, *p *< 0.0001), and were more likely to have 3 or more positive lymph nodes (32.4% vs. 9.1%, *p *< 0.0001). They were significantly less likely to have the ER-positive, PR-positive, HER2-negative subtype (61.5% vs. 82.3%, *p* < 0.002); among the ER positive cases, those with early recurrence were significantly more likely to have PR low tumors (53.9% vs. 31.9%, *p* = 0.002). Patients with early recurrence were also significantly more likely to have grade 3 tumors compared with those without early recurrence (19.1% vs. 2.4%, *p* < 0.0001), have tumors with significantly higher mean Ki67 (21.1% vs. 13.1%, *p* < 0.0001), and have lymphovascular invasion (21.2% vs. 6.5%, *p* < 0.0001). There were no differences in age at diagnosis, menopausal status, or BMI for patients with early recurrence versus those without early recurrence.

Treatment patterns differed significantly in those with early recurrence compared with those without. Those with early recurrence were more likely to receive chemotherapy (52.7% vs. 36.7%, *p* = 0.009), including neoadjuvant chemotherapy (22.9% vs. 12.2%, *p* = 0.013). Patients with an early recurrence were less likely to have had adjuvant endocrine therapy, both overall (60.0% vs. 81.0%, *p* < 0.0001) and among those with ER-positive tumors (69.5% vs. 82.1%, *p* = 0.021). Lumpectomy without radiation was more common in those with early recurrence (n = 19, 55.9%) compared with those without an early recurrence (n = 62, 31.6%) (*p* = 0.006). There was no difference in the mastectomy rates between those with and without an early recurrence.

In a multivariate logistic regression model for early recurrence, stage 3 disease, ER+PR-HER2- receptor subtype, grade 3 tumors, and undergoing lumpectomy alone were all significantly associated with increased odds of recurrence, while adjuvant endocrine therapy use was associated with significantly decreased odds of recurrence (Table 2). Given these associations, we estimated the cumulative incidence of early recurrence for those undergoing lumpectomy alone with or without endocrine therapy, and lumpectomy plus radiation with or without endocrine therapy. There was a significantly higher incidence of estimated cumulative 5-year recurrence among those undergoing breast conserving surgery without adjuvant endocrine therapy (*p* < 0.0001 by log rank). While the estimated cumulative incidence of recurrence in those undergoing lumpectomy with both radiation and endocrine therapy was 3.5% at 5 years, it was 21.6% for those having lumpectomy alone, and 28.6% for those having lumpectomy/radiation without endocrine therapy (Table 3).

**Table 2:** Factors associated with early versus late recurrence, or both, in multivariate analysis

Factors associated with early recurrence only	Factors associated with late recurrence only	Factors associated with both early and late recurrence
Larger tumor size	Younger age at diagnosis	Increased number of positive lymph nodes
Tumor receptor subtype	Higher BMI	PR low status
Higher tumor grade		
Increased tumor Ki67		
Increased LVI		
High risk CTS5 score		
Undergoing lumpectomy alone		
Omission of adjuvant endocrine therapy		
Receipt of chemotherapy

**Table 3:** Estimated 5-year cumulative incidence of recurrence based on local and systemic therapy.

Category	Estimated 5-year cumulative incidence of recurrence (%)	95% CI (%)
Lumpectomy without endocrine therapy	21.6	12.8–35.1
Lumpectomy with adjuvant endocrine therapy	14.2	7.0–27.8
Lumpectomy/radiation without endocrine therapy	28.6	13.0–55.7
Lumpectomy/radiation with endocrine therapy	3.5	1.6–7.6

### Clinicopathologic Features Associated with Late Recurrence

We then compared patients in the “late recurrence” group with those in the “no recurrence” group. Of the 54 patients with late recurrences, 20 patients (37.0%) had local recurrence, 28 (51.9%) had distant recurrence, and 6 (11.1%) had both local and distant recurrence. Late recurrence was significantly associated with younger age at diagnosis (55.7 years compared with 59.2 years, *p* = 0.037), elevated BMI, and more nodal involvement (Table 1). In the late recurrence group, 60.1% had BMI above the normal range compared with 45.4% in the no-recurrence group (*p* = 0.021). While not significant, there were slightly more patients with > 3 positive lymph nodes in the late recurrence group (n = 10, 19.2%) compared with patients without late recurrence (n = 53, 11.8%) (*p* = 0.121). There was no difference in overall stage, grade, or hormone receptor subtype overall. However, among the ER positive cases, those with late recurrence were more likely to be PR low (55.6% vs. 32.6%, *p* = 0.005).

Although not significant, compared with those without any recurrence, patients with late recurrence were numerically more likely to receive chemotherapy (50.9% vs. 37.7%, *p* = 0.061). However, other treatment factors such as type of surgery, delivery of radiation with lumpectomy, and adjuvant endocrine therapy did not differ.

### CTS5 Score

In the 471 patients in the study for whom CTS5 score could be calculated, 34.4% (n = 162) were classified at low-risk, 15.5% (n = 73) were intermediate-risk, and 50.1% (n = 236) were high-risk. Overall, this distribution was similar in pre- and post-menopausal patients. Higher CTS5 score was associated with early recurrence but not late recurrence in this cohort. In those with early recurrence, 66.7% had a high-risk CTS5 score, compared with 47.6% of those without early recurrence (*p* = 0.018). Additionally, high CTS5 score was associated with shorter time to early recurrence (*p* = 0.0109, log rank).

## Discussion

In this study of 513 women with early-stage ILC, we found 75 cases of early recurrence (within 5 years of diagnosis) and 54 cases of late recurrence (5 or more years after diagnosis). We found associations between patient/tumor factors, treatment type, and timing of recurrence.

In general, factors related to increased tumor aggressiveness (such as grade, Ki67, LVI) appeared to be associated with early recurrence, whereas more patient-related factors (such as age, BMI) were related exclusively to late recurrence. Increased number of positive lymph nodes and having low PR expression were significantly more common in both early recurrence and late recurrence cases compared with those with no recurrence. The finding of more aggressive tumor biology being associated with early recurrence is consistent with prior literature.^19,20^

Interestingly, treatment related factors around surgical management and endocrine therapy were associated with early but not late recurrence. For those patients who underwent lumpectomy alone or omission of endocrine therapy, the odds of early recurrence were significantly elevated, even on multivariate analysis adjusting for other factors such as age, receptor subtype, and tumor grade. This finding may inform management strategies regarding adjuvant radiation and/or endocrine therapy. In this cohort, omission of either endocrine therapy or radiotherapy in patients undergoing breast conservation yielded an estimated cumulative recurrence rate at 5 years that exceeded the rate of recurrence seen at 10 years in radiotherapy omission trials such as PRIME-II.^21^ This suggests that omission of adjuvant radiation in the setting of ILC might be associated with higher risk of recurrence than for those with ductal cancers; indeed, lobular histology has been an exclusion criterion for omission or de-escalation trials.^22^ Further research on omission of radiotherapy in patients with ILC is needed.

Regarding late recurrence, we found that younger age, elevated BMI, increasing number of positive lymph nodes, and PR low status were all associated with increased risk. To our knowledge, only one other published study has specifically addressed factors associated with late recurrence in ILC.^14^ Conforti et al. evaluated over 1872 ILC cases for late distant recurrence and found that Ki67 ≥ 20, nodal positivity, and large tumor size were significant predictors of late recurrence. While CTS5 score alone was not associated with late recurrence, the addition of Ki67 led to improved prognostication. In our cohort, we found that higher CTS5 scores were instead associated with early recurrence. We also did not find associations between Ki67 and late recurrence, but Ki67 was unavailable for a large proportion of patients, decreasing the statistical power. Additional differences between our study and the Conforti study include our inclusion of both pre- and post-menopausal patients, as well as the inclusion of local and well as distant recurrences as events. However, we had similar findings regarding nodal positivity, and while larger tumor size was not a significant predictor in our analysis, those with late recurrences trended towards larger tumor size. Our finding of elevated BMI being associated with late recurrence in ILC has not been previously reported to our knowledge; whether this is related to differences in tumor biology related to BMI, versus a pro-estrogenic effect of obesity, or is confounded by other factors such as physical activity which have been shown to impact breast cancer recurrence rates is unknown.^23^

Understanding the timing of recurrence risk has implications for treatment selection and surveillance strategies. For example, patients with shorter life expectancy may be concerned with early but not late recurrence, and may want to consider tailoring treatment based on the potential to impact one versus the other. While we would have hypothesized that endocrine therapy would influence rates of late recurrence in these patients with ILC, we found instead that it was a significant predictor of early recurrence.^24,25^

Because standard imaging studies have decreased sensitivity for ILC, some have advocated for the use of magnetic resonance imaging (MRI) for evaluation of ILC.^26^ While these data address the pre-operative use of MRI, the optimal surveillance imaging strategy for patients with ILC who opt for breast conservation is unknown. The ability to risk stratify patients for early versus late recurrence could potentially help with planning the most useful surveillance imaging plan regarding when to start, frequency, and duration of imaging, areas where there are currently no data in ILC.

While this study utilizes a well-maintained single institution database with reasonably long follow-up, there are many limitations, including its retrospective nature, which result in treatment selection bias. The association between chemotherapy use and early recurrence likely reflects this, as patients with more aggressive tumor features would be more likely to receive a recommendation for chemotherapy. Additionally, we do not have data on duration of endocrine therapy use, nor on surveillance strategies. However, these real-world data may have increased generalizability as they reflect real-world conditions.

In conclusion, we identified factors that may help stratify patients at high risk for early or late recurrence after treatment for early stage ILC. Further data are needed, especially regarding the safety of omitting treatment such as radiation or endocrine therapy in patients with ILC, and in the area of surveillance strategies.

## Data Availability

The data supporting all Tables in this published article are not publicly available to protect patient privacy, but can be accessed from the corresponding author on request. Data will be made available to authorized researchers who have obtained Institutional Review Board (IRB) approval from their own institution and from the University of California, San Francisco IRB.
